# In spite of the system: A qualitatively-driven mixed methods analysis of the mental health services experiences of LGBTQ people living in poverty in Ontario, Canada

**DOI:** 10.1371/journal.pone.0201437

**Published:** 2018-08-15

**Authors:** Lori E. Ross, Margaret F. Gibson, Andrea Daley, Leah S. Steele, Charmaine C. Williams

**Affiliations:** 1 Dalla Lana School of Public Health, University of Toronto, Toronto, Ontario, Canada; 2 School of Social Work, York University, Toronto, Ontario, Canada; 3 Department of Family and Community Medicine, St. Michael’s Hospital, Toronto, Ontario, Canada; 4 Factor-Inwentash Faculty of Social Work, University of Toronto, Toronto, Ontario, Canada; ESIC Medical College & PGIMSR, INDIA

## Abstract

Lesbian, gay, bisexual, trans, and/or queer (LGBTQ) people face barriers to accessing mental health care; however, we know little about service experiences of low income LGBTQ people. In this qualitatively-driven mixed methods study, over 700 women and/or trans people completed an internet survey, of whom 12 LGBTQ individuals living in poverty participated in interviews. Low income LGBTQ respondents saw more mental health professionals and had more unmet need for care than all other LGBTQ/income groups. Narrative analysis illustrated the work required to take care of oneself in the context of extreme financial constraints. These findings highlight the mechanisms through which inadequate public sector mental health services can serve to reproduce and sustain both poverty and health inequities.

## Introduction

Lesbian, gay, bisexual, trans, and queer (LGBTQ) populations, relative to non-LGBTQ populations, experience significant health disparities [1] particularly in relation to mental health. Data from a number of meta-analyses consistently indicate that sexual minority people have higher rates of a variety of mental health problems, including depression, anxiety, and suicide, relative to their heterosexual counterparts [2–3]. Although fewer data are available regarding trans mental health, as a result of the invisibility of gender identity in most population-based surveys, available evidence suggests that mental health disparities are even more pronounced for trans people relative to cisgender (non-trans) people [4].

There are also important economic disparities associated with sexual orientation and gender identity. For example, Badgett and colleagues [5] examined data from three different US population-based surveys, and found a consistent pattern of elevated poverty rates among sexual and gender minority people. These findings align with data from other countries, including Canada [6] and the United Kingdom [7], which have also identified disparities associated with LGBTQ identity on a variety of economic indicators. Further, the data align with evidence from general population surveys showing that economic disparities intersect with other variables such as race, gender, and geography (e.g., elevated poverty rates among female same-sex couples, among Black members of same-sex couples, and among same-sex couples living in rural areas, relative to other sexual minority people [8].

Although there are well-established relationships between poverty and health [9] and LGBTQ identity and health [2], few studies have considered how LGBTQ identity and poverty may operate in concert to determine health. One exception is a quantitative study by Veenstra [10], in which he examined Canadian population-based data to explore inequities in self-reported health associated with various axes of marginalization, including sexual orientation and poverty. He found that self-reports of poor health were more common among individuals who reported their sexual orientation as ‘homosexual’ and who reported low income, in comparison to both ‘homosexual’-identified individuals without low income, and to heterosexual individuals with low income [10]. Further, he found that the difference between groups was of a greater magnitude than would be expected based on the effect sizes for the independent effects of sexual orientation and low income on this outcome [10].

In previous quantitative research by our team, we have similarly examined the impact of multiple marginalized statuses on health-related outcomes; specifically, on depression and unmet need for mental health care among women and gender liminal (trans) people [11]. Results of this analysis of survey data revealed that low income and gender liminal status both operated independently and in interaction with racial minority status to predict depression and unmet need for mental health services. However, interaction terms between low income and sexual minority or gender liminal status were not significant in either model. Further, experiences of discrimination were the most proximal predictor for both outcome variables, suggesting that discrimination, rather than the specific identities that elicit discrimination, is likely the root cause of disparities in mental health and health service access [11].

Taken together, these quantitative studies suggest that important contextual factors, including gender and experience of discrimination, may be critical to understanding the health service experiences of low income LGBTQ people. Traditional quantitative methods limit our capacity to understand contextual factors: regression approaches, as used by both Veenstra [10] and in our team’s prior work [11], permit examination of the role of a single factor (or at most, two or three factors in interaction) while holding all other measurable factors constant. While this approach has aided in identifying factors worthy of additional investigation, it oversimplifies the relationships between factors, and thus tells us little about their complex inter-relationships.

### Theoretical approaches

We situate this work within a transformative paradigm, wherein a plurality of research methodologies can be combined with the uniting goal of achieving social justice [12]. In this case, we worked in collaboration with LGBTQ community members and agencies towards addressing inequities experienced by LGBTQ people in their attempts to access mental health care [13]; the present analysis focuses specifically on inequities experienced by LGBTQ people living in poverty. Towards this end, our primary guiding theoretical framework was intersectionality theory, as articulated by legal scholar Kimberlé Crenshaw [14]. Drawing on the work of other Black feminist scholar/activists (e.g., Davis [15]), Crenshaw coined *intersectionality* to explicate the specific experiences of Black women being denied claims of employment discrimination [14]. Intersectionality theory attends to how forms of structural oppression intersect and are mutually reinforcing, resulting in, as Patricia Hill Collins [16] described, a matrix of domination. Although initially applied to the experiences of Black women in particular, intersectionality has since been applied to others who embody intersecting oppressions, including sexual minority women [17], and to other contexts, including health care [18].

We drew upon intersectionality theory to guide our sampling, data collection, and analysis. Specifically, we structured our quantitative sampling to ensure the inclusion of adequate representation of individuals at each of the pertinent intersections (i.e., LGBTQ people living in poverty, cisgender heterosexual people living in poverty, LGBTQ people not living in poverty, and cisgender heterosexual people not living in poverty). This enabled us to investigate not only experiences at the intersections of oppressed identities/experiences, but also to investigate experiences at the intersections of privilege. Attention to intersectionality in our data collection and analysis processes principally entailed attention to experiences of discrimination and privilege, and recognition of the inextricable nature of our participants’ experiences of their sexual orientation, gender identity, and social class.

In applying intersectionality theory through the lens of a transformative paradigm, we have drawn upon what McCall [19] has termed both intercategorical approaches (in our quantitative analysis) and intracategorical approaches (in our qualitative analysis) to intersectionality. That is, in service of our transformative (i.e., social justice) goals, we have strategically essentialized categories associated with sexual orientation, gender identity, and poverty in our quantitative analysis [11], while simultaneously recognizing these categories to be both dynamic and socially constructed in our qualitative analysis. Our specific approaches to the construction of each of these categories are described in detail below.

### The current study

In order to address this research gap, the current qualitatively-driven mixed-methods study aims to answer the following primary research question: *what are the mental health service experiences of Ontario sexual minority women and/or trans people living in poverty*? Within this, our quantitative strand addresses the sub-question: *how do these experiences compare to those of individuals of other sexual/gender identities and who are not living in poverty*? Finally, our qualitative strand addresses the following sub-question: *how might the relationship between LGBTQ identity and poverty operate to explain these findings*? Investigation of this primary research question and sub-questions will provide a contextualized account of mental health service experiences at the under-studied intersection of LGBTQ identity and poverty.

## Methods

### Study design

The present study describes a reuse of data [20] from a community-based, mixed methods study for which the first author was the principal investigator. The parent study examined experiences with depression treatment among women and/or trans people in Ontario, Canada with a focus on understanding differences associated with sexual orientation and gender identity [13]. The study utilized a sequential explanatory mixed methods design [21], wherein all participants completed an internet-based survey and, based on their responses to survey questions about their experiences of depression over the past 12 months, a subset of participants were purposefully selected for recruitment into the qualitative strand (semi-structured qualitative interviews, as described below). The study was reviewed and approved by the Research Ethics Board of the Centre for Addiction and Mental Health, Toronto, Canada.

The reuse of data described in this manuscript is a qualitatively-driven mixed methods analysis [22], where we used an iterative approach to re-examine both the qualitative and quantitative data with new research questions about mental health service experiences for LGBTQ people living in poverty. Although the term “qualitatively-driven mixed methods” was introduced by Mason [22] over a decade ago, it has not been well characterized in the literature. While it might be interpreted to refer to mixed methods designs utilizing a qualitative strand as the priority strand [21], it has also been interpreted to refer exclusively to mixed methods designs wherein a core qualitative strand is presented first in sequence [23]. However, Mason’s [22] initial writing defined the term to refer to mixed methods research wherein qualitatively-oriented approaches to epistemology (e.g., social constructionist worldviews) and methodology (e.g., interpretive approaches) are applied to quantitative data, and it is this definition that we applied in the current study.

Specifically, the first author came to formulate questions about the role of poverty in her process of conducting qualitative interviews for the parent study. An initial set of descriptive questions were tested against the quantitative data (to identify any differences in mental health service experiences between groups constructed on the basis of LGBTQ and low income status), and results of this preliminary testing informed a first pass at qualitative data analysis (i.e., immersion in the qualitative data with particular attention to segments of each transcript that alluded to topics related to socioeconomic status, income, or poverty). The first author then continued to move between the quantitative and qualitative data in an iterative process until the final set of research questions (as stated above) had been first formulated, and then answered.

For the qualitative strand of this reuse of data, we utilized a thematic narrative analysis as described by Riessman [24]. Narrative analysis is a qualitative methodology that is commonly applied in the social sciences [24], but has seldom been used as part of a mixed methods study design (for what is to our knowledge a lone example, see [25]). Thematic narrative analysis is similar to other forms of thematic analysis, in that the analyst searches for common themes in the data. In narrative thematic analysis, however, “narrative scholars keep a story ‘intact’ by theorizing from the case, rather than from component themes (categories) across cases” (p. 53) [24]. Although the primary qualitative data for the parent study were not conducted with narrative analysis in mind, after our initial pass at qualitative analysis, we determined that a narrative approach would be most appropriate. The data relevant to our research questions were not organized into discrete chunks of text, nor always discussed explicitly, but rather tended to infuse the entire ‘story’ of mental health service access as told by participants. As such, our unit of analysis for the qualitative strand was the complete interview transcript (“case”).

### Recruitment and participants

Recruitment methods have been described in detail elsewhere [13]. Briefly, we used targeted convenience sampling, wherein we recruited anyone who identified as a woman and/or trans person via advertisements distributed electronically and in hard copy throughout LGBTQ- and mental health-oriented programs and services across the province of Ontario, Canada. Advertisements invited participation in a study of “depression treatment in Ontario”. In order to ensure that adequate numbers of typically underrepresented groups (i.e., low income, racialized, trans and bisexual people) were reached, we first targeted recruitment to these individuals through relevant programs, services, and electronic listserves; research staff also visited key programs in person to provide information and access to research materials. Recruitment was expanded more broadly once target numbers for each of these groups had been met.

To be eligible to participate, individuals had to live in Ontario, be 18 years of age or older, be sufficiently fluent in English to understand the research materials, and self-identify as a woman and/or trans person (including, but not limited to, self-identities such as transgender, transsexual, genderqueer, and person of trans experience).

### Survey

Quantitative data collection occurred between June 2011 and July 2012 via internet survey, as described in detail elsewhere [13]. Hard copies of the survey were also available upon request to facilitate participation of individuals without private internet access, though all participants ultimately completed the survey via internet, either independently or during data collection visits at relevant community programs (e.g., weekly drop-in program for LGBTQ people at a community mental health agency).

Details of all survey items utilized in this analysis are provided in Appendix A. In brief, items to assess sexual orientation and gender identity were based on a previous pilot study [26] and revised with input from our community advisory committee. Participants who selected only “heterosexual” for sexual orientation and “woman” for gender identity were coded as cisgender heterosexual; all other participants were coded as LGBTQ. Poverty was operationalized as being below the low-income measure (LIM), which reflects 50% of the median household income adjusted for household size [27]. Items to assess the dependent variables were developed on the basis of the Canadian Community Health Survey, a population-based survey of the health of Canadians, Cycle 1.2: Mental Health and Well-Being [28].

Survey participants who chose to provide contact information received a $10 gift card as compensation; those who wished to participate anonymously had the option of directing their compensation to a registered charitable organization.

### Interviews

For the parent study, one of the authors or a trained research coordinator conducted 24 semi-structured qualitative interviews, averaging 84 minutes in length (range: 52 min– 151 min), between June 2012 and January 2013. Potential interview participants were selected from the pool of survey respondents who indicated their willingness to be contacted for a follow-up interview. Of the respondents who were willing, those who were LGBTQ-identified were contacted for a brief telephone screening interview, during which their past 12 months depression status was assessed using a structured tool, the Composite International Diagnostic Interview [29]. Participants who screened positive for past 12 months depression were then asked to provide some sociodemographic information and to provide a brief description of their mental health service experiences over the past year. On the basis of this information, the 24 participants were purposefully selected for interviewing to achieve a maximum variation sample [30] on the basis of relevant characteristics such as sexual and gender identity (lesbian, bisexual, trans); racialized identities (Indigenous, non-Indigenous racialized, white), and socioeconomic status, and well as on the basis of extent of mental health service utilization (ranging from limited to no service engagement to extensive engagement in the prior year).

For the present analysis, 12 of the 24 interviews were selected for analysis. Initial selection was made on the basis of below LIM status; however, examination of the qualitative data revealed that this widely used index was imperfect in capturing experiences of poverty. Data from the qualitative interviews were then used to re-select interview cases that would best achieve our analytic goals (i.e., using the LIM as a starting point, but supplementing with an additional case whose interview provided rich data about the impact of poverty on her experience; cases that met the LIM criteria but were not experiencing sustained poverty to the same extent as other included participants were treated as negative cases [31], against which we could compare and contrast the identified themes).

Interviews flexibly followed an interview guide developed by the researchers to address the primary qualitative research question for the parent study (“what are the barriers to effective depression treatment encountered by lesbian and bisexual women and/or trans people in Ontario?”). The interview guide was field-tested with members of the community advisory committee and research team who identified with the participant groups (i.e., as lesbian, bisexual, or trans).

The interview guide opened by asking participants to share their experiences of the past year as they relate to their mental health, with particular emphasis on understanding the context of the participant’s recent life experience and its relation to depression and/or other mental health issues. On the basis of the response to this question, the interviewer went on to explore any experiences of accessing or attempting to access mental health services during the past year. If services had been accessed, the participant was asked a number of follow up questions to characterize their experiences with that service (e.g., “Can you tell me about a specific experience with [service]?” “What made you feel comfortable/uncomfortable?”). If the participant had not accessed mental health services in the past 12 months, they were asked follow up questions regarding their decision not to access services and/or any barriers they encountered in their attempts to access services.

Following the interview, participants received a cash honorarium, as well as a list of mental health and LGBTQ community resources.

### Data analysis

#### Quantitative analysis

Four mutually exclusive groups were constructed on the basis of participant categorization as LGBTQ vs. cisgender heterosexual and below vs. above LIM, as described above. Bivariate analyses (chi-square tests or analysis of variance as appropriate) were then used to compare the four resulting categories, first on the basis of sociodemographic variables, and finally on the basis of the mental health service utilization variables. Post-hoc tests were conducted with Bonferroni corrections for continuous variables, and adjusted standardized residuals (ADJR) were computed for categorical variables. ADJR values indicate the relative contribution of each cell to a significant chi-square test, with values >1.96 and <-1.96 indicating significantly higher or lower (respectively) observed proportions than would be expected by chance for a 2 x 2 table; for tables with larger cells, values of >3 or <-3 are recommended to account for risk of Type 1 error [32]. SPSS version 23 was used for all quantitative analyses.

#### Qualitative analysis

As noted above, qualitative data were analyzed using a thematic narrative analysis approach [24], wherein each interview transcript was treated as a unit of analysis, and themes were identified across these units. After initial immersion in the data set as a whole, the first author reviewed each of the 12 selected transcripts in detail, noting any segments that explicitly or implicitly referenced poverty, social class, or other relevant topics. Within each interview, she then more closely examined these segments, making note of how they collectively told a story about the participant’s experiences of accessing mental health care, as well as meeting any mental health needs that were not met by the formal mental health system. The second author then reviewed these notes, together with the transcripts, and added her own analytic comments.

Through this process, the first two authors together developed the core theme, ‘the work of taking care’, and re-examined the transcripts to ensure fidelity with this core theme. The first author then went back to the transcripts and analytic notes to identify the four subthemes (types of ‘work’ done by our participants in taking care of themselves and others), and collected together interview segments to illustrate each of these four subthemes. As a final step, all of the coauthors, who had been deeply engaged with the research data as part of the primary study, reviewed the presentation of the findings for the purposes of peer review/debriefing [31]. The final version of our analysis, incorporating feedback from the peer debriefing process, is presented here.

#### Mixed methods analysis

Mixing was continuous throughout the data analysis process, with findings from each strand informing the ongoing analysis of the other. In the presentation of results below, we present first the final version of the quantitative analysis, followed by the final version of the qualitative analysis. We then offer an integrated interpretation of the results of the two strands in the Discussion section.

## Results

### Quantitative findings

A total of 704 eligible participants completed at least some of the survey, of whom 696 (98.9%) provided sufficient data to be classified on the basis of LGBTQ/LIM status. Of these, 222 (31.9%) were LGBTQ-identified and living below the LIM (LGBTQ/LIM), 231 (33.2%) were LGBTQ-identified and living above the LIM (LGBTQ/not LIM), 110 (15.8%) were cisgender heterosexual and living below the LIM (not LGBTQ/LIM), and 133 (19.1%) were cisgender heterosexual and living above the LIM (not LGBTQ/not LIM).

Sociodemographic characteristics of the four groups are provided in Table 1. There were statistically significant differences in all of the variables examined. Both cisgender heterosexual groups were significantly older than the LGBTQ groups (41.3 and 42.2 years vs. 34.7 and 36.8 years, respectively; F = 16.4, p<0.001). The LGBTQ/LIM group disproportionately lived in Toronto, Ontario’s major urban centre (60.5%), while the not LGBTQ/not LIM group disproportionately lived outside of Toronto (33.6%, χ^2^ = 28.4, p<0.001). As would be expected, participants with high school or less education were over-represented in both LIM groups (47.7% and 43.5% of the LGBT and not LGBT groups, respectively; χ^2^ = 79.9, p<0.001), while participants who were full-time or self-employed were under-represented in these groups (14.7% and 20.5% of the LGBT and not LGBT groups, respectively; χ^2^ = 171.2, p<0.001). Indigenous participants were over-represented in the not LGBTQ/LIM group (17.0%) and under-represented in the LGBTQ/not LIM group (7.3%; χ^2^ = 8.6, p<0.05). In contrast, white participants were over-represented in the LGBTQ/not LIM group (71.6%; χ^2^ = 8.2, p<0.05).

**Table 1:** Sociodemographic characteristics by LGBTQ and low income measure (LIM) group (N = 696).

	LGBTQ/ LIM (n = 222)	LGBTQ/ Not LIM (n = 231)	Not LGBTQ/ LIM (n = 110)	Not LGBTQ / Not LIM (n = 133)
Mean Age (SD)	34.7 (11.7)	36.8 (10.3)	41.3 (12.5)^†^	42.2 (10.9) ^†^
Live in Toronto** ADJR	60.5% 3.5	54.9% 1.6	41.9% -1.9	33.6% -4.3
High school or less education** ADJR	47.7% 6.9	18.1% -4.9	43.5% 3.3	11.3% -5.3
Full-time or self-employed** ADJR	14.7% -9.9	61.2% 7.4	20.5% -5.0	71.2% 7.6
Indigenous identity* ADJR	12.8% 1.1	7.3% -2.2	17.0% 2.2	9.0% -0.8
White identity* ADJR	61.5% -1.5	71.6% 2.4	58.0% -1.8	66.9% 0.4

LGBTQ: lesbian, gay, bisexual, trans, and/or queer; LIM: below the low income measure; ADJR: adjusted standardized residual

^†^ indicates a statistically significant difference from the LGBTQ/LIM group at p<0.001

* indicates a significant chi-square test at p<0.05

**indicates a significant chi-square test at p<0.001

Comparisons between the four groups on mental health service utilization variables are provided in Table 2. In total, 444 participants provided sufficient income and mental health service utilization data to be included in the primary analyses. Again, there were statistically significant differences between groups in all the variables examined. With respect to access to mental health care, both LIM groups reported a higher proportion of participants having accessed care in the past 12 months, with the highest rates of access in the LGBTQ/LIM group (68.3%, compared to 51.2% in the most privileged–not LGBTQ/not LIM–group; χ^2^ = 11.3, p = 0.01). Similarly, LIM groups reported seeing more professionals in the past 12 months than did not LIM groups, again with the highest number in the LGBTQ/LIM group (a mean of 3.24 professionals compared to 2.14 in the not LGBTQ/not LIM group; F = 6.54, p<0.001). However, despite these high rates of access, the LGBTQ/LIM group also reported the highest rates of unmet need for mental health care (77.8%), while the not LGBTQ/not LIM group reported the lowest rates (51.2%; χ^2^ = 16.9, p = 0.001). Similarly, the LGBTQ/LIM group reported the lowest proportion of professionals seen in the past 12 months who were helpful (0.55), while the not LGBTQ/not LIM reported the highest proportion (0.73; F = 5.47, p = 0.001).

**Table 2:** Mental health service utilization in the past 12 months by LGBTQ and low income measure (LIM) group (N = 444).

	LGBTQ/ LIM (n = 158)	LGBTQ/ Not LIM (n = 149)	Not LGBTQ/ LIM (n = 65)	Not LGBTQ/ Not LIM (n = 72)
Accessed care in past 12 months* ADJR	68.3% 2.9	56.3% -1.4	62.3% 0.5	51.2% -2.2
Mean number of professionals seen (SD)	3.24 (2.75)	2.23^†^ (1.55)	2.79 (1.83)	2.14^†^ (1.63)
Unmet need for mental health care** ADJR	77.8% 3.6	68.3% 0.1	61.3% -1.7	58.1% -2.7
Proportion of professionals seen that were helpful (SD)	0.55 (0.36)	0.70^†^ (0.36)	0.69 (0.35)	0.73^†^ (0.36)

LGBTQ: lesbian, gay, bisexual, trans, and/or queer; LIM: below the low income measure; ADJR: adjusted standardized residual

^†^ indicates a statistically significant difference from the LGBTQ/LIM group at p<0.01

* indicates a significant chi-square test at p<0.05

**indicates a significant chi-square test at p<0.001

### Qualitative findings

Across all interviews, the stories that our participants told about attempting to access mental health services were structured around a common theme of *work*. Aligning with our quantitative findings in relation to unmet need, participants described working hard to find, or to create for themselves, the supports they needed to take care of themselves (and often others) in the face of multiple, intersecting forms of structural oppression. That is, participants described working hard to take care of themselves in spite of, rather than with the support of, the formal mental health system.

Participant narratives recounted four central kinds of work:

- strategizing to overcome oppression encountered in processes of employment and employment seeking
- actively researching and seeking out services that could meet their specific intersectional needs within a budget they could afford
- responding to and coping emotionally with past and contemporary experiences of violence, trauma, and microagressions, including from service providers;
- creating supports in order to meet needs that the system failed to address.

While not every interview in our data set provided evidence in support of all four of these subthemes, each subtheme was apparent, at minimum, across several interviews. Specifically, the first subtheme, employment, was prominent in the narratives of those participants who were working or were actively seeking work, but was less apparent in the narratives of those participants who were unable to work due to their mental health or parenting responsibilities. The second subtheme, access to services, was universal across narratives, particularly considering that in the Ontario context, much of mental health care is delivered in the private sector. With respect to the third subtheme, experiences of discrimination and microaggressions were discussed in all of the interviews, though participants did not universally attribute their experiences in this way. Experiences of violence or trauma were not universal; however, they were described in several of the interviews, and played a central part in these participants’ narratives. Finally, the fourth subtheme regarding creating supports was universal across all narratives, in the context of identifying supports for themselves or others in their immediate circle outside of the formal mental health system. In addition, for a subset of the narratives, this theme captured the work of building resources at the community level, wherein participants took to initiative to develop resources that would benefit people outside their immediate personal network. Across all four forms of work, participants demonstrated enormous agency, creativity, and resourcefulness, maneuvering within the constraints of intersecting classism, ableism, racism, misogyny, and heterosexism/monosexism/cissexism to leverage often scant practical and emotional resources to take care of themselves, their families, and their communities.

In order to illustrate these subthemes we share the narratives of four of the participants whose stories are particularly illustrative. All names are pseudonyms.

#### Kirk’s story: “I don’t feel useless anymore”

Many of our participants talked about how employment affected both their mental health and their experiences accessing mental health services. Employment could be a source of positive mental health when participants were able to secure a stable job in an LGBTQ-affirming workplace. More often though, employment (or the lack thereof) was a source of stress, whether due to precarious and difficult working conditions, because of actual or feared employment discrimination, or because of the stress of unsuccessfully searching for work. Challenges related to employment directly affected participants’ mental health service experiences: when work was a source of stress, they experienced greater need for mental health services, but when participants could secure a stable job with benefits, they had greater access to LGBTQ-affirming mental health supports.

Kirk’s story illustrates some of these themes. Kirk is a trans man of East Asian descent who was questioning his sexual identity at the time of the interview. Kirk was in his early twenties at the time of the interview, had graduated from college about a year prior, and had spent most of that year trying to find work.

My expectation was that, after I graduate—and I was getting pretty good marks too—I thought I would get a good job, or any job possible. And I even travelled to [name of city five hours from Kirk’s home] to go to an interview and job test there, but nothing really worked out. And then I, I go on Facebook and Twitter and I see my friends and my peers getting the jobs. Well, not everybody but at least a lot of the people that I knew closely were getting jobs. So, I was feeling very depressed at that time… So, basically I was doing nothing and I was feeling very useless and I really didn’t feel good at all.

For Kirk, not being able to find work had not only emotional consequences, but also practical consequences: it meant that he had to move back with his parents. This meant re-exposing himself to an environment that was very challenging for his mental health because of his parents’ transphobia:

Now, I’ve learned how to cope with certain things about my parents, but since I came out three years ago it was pretty bad I guess. They weren’t really, like, oppressive but because they are Asian and they are very conservative Christian they had this, how do you say it, silent ways of oppressing. They are more like, I don’t hear anything, I don’t see anything, I don’t say anything, that kind of stuff. So, that’s a bit of a challenge, it’s still like that.

Kirk’s path to dealing with his depression included volunteering with a group for LGBTQ Asian youth, where he was not only able to find social support, but also make use of his professional skills:

I volunteered to make some kind of poster for them and they really liked it. And that was a big deal to me because at that time I didn’t have any job to kind of use my skill, and there they kind of acknowledged it.

Kirk described this time–when he was able to use his skills through his volunteer position–as a turning point for his mental health. Ultimately, he was able to find a job through a connection he made in the youth group, although it was in a different field than the one in which he had trained. When asked how it felt to be working, Kirk replied:

It’s good, yeah, now I don’t feel useless anymore. I recently thought, you know, I’m dipping myself into a bit too many things and I kind of got a chance to reflect why I was doing this, and it kind of links back to how I was last summer. Like, I don’t really want to feel like I’m useless, like I’m not doing anything anymore.

Kirk never raised the possibility that racism or transphobia might have factored into his employment struggles, but his story echoes the stories of many of our participants who worked hard to search for employment in the face of discrimination. For Kirk, and other participants, not being able to find employment led to feelings of uselessness and low self worth, in addition to very practical challenges associated with lack of income, such as safe housing and food insecurity. These stresses collectively created a need for mental health supports, but as the next story will illustrate, these needed supports were not easily accessible.

#### Rachel’s story: “On my own initiative”

Rachel is a white, Jewish bisexual cisgender woman who lives in a wealthy suburb of a major urban centre in Ontario. She was in her mid-thirties at the time of her interview, and living in a small apartment with her husband and their school-aged child. Rachel had struggled with her mental health since childhood, and particularly with suicidal ideation and severe anxiety, which at times made it impossible for her to leave the house.

Like many of our participants, Rachel had developed a very nuanced strategy for getting her needs met when her mental health “crashed” (to use her word). Central to the success of this strategy was her own self-awareness and her ability to mobilize informal and formal support networks. Rachel described her process this way:

Generally, I’d say I have a crash about once a year, once every 2 years, now. As long as I’m taking my meds. As long as I can reduce the stress over time, I’ll be fine. If I don’t have the ability to either change my environment, which is when I go to [respite service], or I don’t have access to my meds, or I don’t have the supports for some reason, that’s when it gets difficult. Luckily enough, I have enough back up supports that that rarely happens.

Rachel’s narrative provides a rich example of the work that goes into finding appropriate and affordable mental health services. Over the course of our interview, Rachel explained that she had needed to work within systems to get her needs met from an early age, as part of her childhood experiences of poverty and foster care. Private sector mental health services were completely out of reach for Rachel, so she had identified a variety of free or publicly funded services that could meet her needs. In our interview, she named off several different services available in her region, and knew every detail of the scope of their services, admission requirements, wait lists, and even the number of sessions she could access from each.

Even making use of all that was available through the public sector mental health system, however, Rachel experienced significant gaps. For example, all of the services she had access to addressed acute needs or were short-term in nature. Only her family doctor offered ongoing mental health support, and this was limited to refilling her prescriptions. However, Rachel struggled with being able to afford these medications. Her husband worked irregular hours, which meant that their income came in bursts, a pattern that created problems for the prescription support programs Rachel needed:

Because I’m on a disability pension, I can get my meds when I have my disability coverage, but…Sometimes, I’m not covered by disability… but I can’t not have my meds, and there is no middle ground. So it comes and goes, and that’s really difficult to manage sometimes, especially because we don’t know, month to month, whether we’re going to be covered.

Rachel also spoke directly to the impact of discrimination from health care providers on her access to mental health care. For Rachel, this was discrimination associated with her mental health history, her poverty, her bisexuality, the fact that she and her husband had an open relationship, and intersections of all of these aspects of her experience. For example, she talked about her sense that her bisexuality and polyamory were not things she could talk about with her care provider:

Interviewer: And did you try to broach it?

Rachel: I attempted to, a little bit, because [partner] is one of my main supports, [ex-partner] is one of my main supports. But. [sigh]

Interviewer: And what kind of response did you get when you tried to bring it up?

Rachel: Basically, ‘I don’t want to talk about that.’ And it’s like, ‘Well, then don’t ask me who my support system is. I know it’s your job to ask me who my support system is. But then don’t judge me on that support system. My support system works for me.”

Rachel’s narrative of discrimination in health care described how her experiences of poverty and her mental health history intermingled in providers’ responses to her:

What I find here in [suburb] is that if you are on OW or ODSP [provincial income supports], you are automatically considered to be a drug addict. You are automatically considered to be worthless. Like, we’ve been over at the hospital…So when [child] was born, we actually had the social workers literally going ‘I don’t know about this, you have mental health issues,’ and it’s like, that doesn’t mean I’m going to be a bad parent! Right?

Rachel, like many of our participants, showed remarkable resourcefulness in filling in the gaps in the formal support system with informal services she pulled together herself. For example, Rachel felt that what would be really helpful for her agoraphobia would be to have someone come check on her periodically, to support her in leaving the house. She described her process of trying to find that kind of service, saying:

Most of what has happened as far as treatment in the last 10, 12 years, has been on my initiative. Without that initiative, I’d still be a mess, but being able to say ‘Ok, I think I need’- and then I go looking for it, and if it’s not in my already set-up plans, then I’ll go and look for it. And that’s where I tried to contact [various services], but they don’t do that thing where somebody comes in and just checks on you. They don’t do it. So I’m like, ‘Ok, well what can I do?’ So now I have a group of friends who will come over, and if I call them, they’ll be over. You know? It’s the best thing I can do. We’re lucky in that sense, that we have those people, but not everybody does.

#### Jacinda’s story: “I’ve just learned how to soldier on”

While discrimination from mental health and other service providers was a common theme across interviews, Jacinda’s story perhaps most vividly illustrates how the oppression associated with marginalized identities is enacted in very real and harmful ways in mental health service settings. Further, Jacinda’s story illustrates the ways in which violence and life-long poverty are deeply interconnected for some low income (and particularly racialized) LGBTQ people.

Jacinda is an Indigenous, bisexual, cisgender woman who was in her mid 30’s at the time of the interview. She was living in a rural Ontario town with her preschool aged daughter. Jacinda described struggling with depression and also suspected other diagnoses were at play, though these had never been confirmed. Jacinda explicitly linked her mental health struggles to her experiences of childhood trauma, intersecting with her adulthood experiences of poverty:

I’ve always had low-level depression. I’ve had a horrific upbringing…So I’m always striving for optimal health, but it’s a struggle ‘cause then there’s other things that sort of get in the way. Like, I’m constantly stressed out about money, right. Constantly stressed out about, like, am I going to make it this month, and budgeting, oh my god, I have no concept of budgeting or money or anything…So, I don’t know what it feels like to not have depression, to not have PTSD, like if somebody, for example, were to come into my house and start yelling in my general direction, I would want to curl into a tiny ball and go into a corner. Or something. I’d need to remove myself ‘cause it triggers me in a big way. Or if somebody starts clinking beer bottles around in a big way, it makes me cringe inside. Like, to normal people, clinking beer bottles in a box just sounds like that, but to me it makes me feel like something horrible is about to happen, right?

For Jacinda, education and employment both required extensive work in order to cope with experiences of violence, poverty, discrimination, and struggles around mental health. She said, “I struggled, I fought tooth-and-nail through my BA” and described having been involved in sex work to support herself and her daughter in the process. At the time of the interview, she was relying on financial support from her co-parent (a queer man who is not her daughter’s biological father) and an “unofficial adoptive father” (an older white heterosexual man who had provided her with financial and practical support) to make ends meet. Jacinda connects feelings of financial dependency, intersecting with feelings of isolation and a desire to be a better parent, to her decision to try to access mental health services:

I was experiencing a lot of hopelessness and depression and just feeling extremely powerless. Because I was just living in, well, essentially a rich man’s house, but not having my own space, not being able to make my own decisions. Um, just feeling pretty dependent and helpless … So I asked [unofficial adoptive father], like I said he’s very affluent, I said ‘would you mind if I went to go see a therapist?’ And he’s like ‘Yeah, okay, well, you really need it. Because I want you to stop drinking and I really would love for you to try and be..’..I wanted to try and be a better parent to [daughter]. ‘Cause if I didn’t live in that space with him, with that support, I don’t know if CAS [child protection] would’ve been knocking on my door or what. I have no idea, ‘cause I just wasn’t in a good space. And not to mention I wasn’t feeling connected to the community at large, ‘cause [small town] is really conservative. It’s very, it’s a sea of milk—that’s how I describe it. And everyone seems to be affluent and heterosexual and it’s like, wow, where is my place here? There is no place.

Jacinda’s first and last purposeful encounter with a mental health professional occurred in the context of this community where her Indigenous, bisexual, and class identities were not only invisible, but ultimately sites of violence:

So I made my appointment and I went there. And he, like, right from the get-go, I don’t think his methodology was very professional at all. Like, he sat me down for four minutes, gave me a booklet that contained a battery of tests, left me alone in a room to fill out this test and then didn’t tell me that he wasn’t gonna be back to debrief or anything. And then he essentially left for the day. I’m not even kidding you…And then we came to his office and—I know I dress suggestively. I don’t wear a lot of clothes and I wear short skirts and I’m comfortable in my own body and this and that. But that should be neither here nor there. You’re here in a professional capacity. I’m here asking for help. Not only that, I’m paying you $200 an hour. Right? So that’s a lot of money… So, he’s sitting there. And I can tell that he’s looking me over. And he’s, sort of trying to gauge where I’m at and what I want. And he said ‘you know, if you don’t feel comfortable in my office Jacinda, I have a practice in my home in [nearby municipality]’. I’m like ‘oh, I love [nearby municipality], it’s one of my favourite places’. And he’s like ‘that’s great, so why don’t you come to my house, you know, let’s say Tuesday’ or whatever it was ‘and we’ll talk further’. And I’m like ‘okay’. I’m thinking finally I’m gonna get some help. Like, praise Jesus, I need some effing help because I feel like I’m not myself, totally depressed and isolated. And so I come to his house and I’m not even lying to you at all, he answers the door, like, totally casually dressed, with a glass of red wine in his hand. How does he know that I don’t have substance abuse issues? He’s got a glass of wine in his hand. And I’m, like, you could be triggering the heck out of me, you don’t know that? Proceeds to sit down next to me on a white leather couch and, like, just chats with me with like there’s no boundaries. And finally I’m so uncomfortable after around ten minutes I, like, excused myself. He still billed me for that session…He was there to see if he could get laid. I’m not even kidding you. That was, that was it. I was, like, okay, well, that’s nice. So that was my experience with attempting to access mental health services.

Jacinda never made any formal complaint about this encounter, noting “it’s amazing when you can get out of bed sometimes when you’re depressed, let alone, you know, file an angry complaint with the college or whatever.” Still, this experience made it difficult for her to approach other providers, particularly in the context of her small community. She contrasted her current situation to a larger community where she had previously lived:

It’s such a close community, I’ve been told not to share anything [with a mental health provider] because it’ll be spread to the larger community. And my [adopted] dad is a respected member of the community. He owns a lot of properties. He knows a lot of people and it would damage his reputation. Because, like I told you, I’ve been a sex trade worker. I’ve been through a lot of experiences that these people would not be able to–I don’t think they would even know what to do with that information…For example, in [city in another province], where I’m from, I knew that there were services and avenues for help, right. Like, I could go, for example, to the Aboriginal Centre where they had a healing center. So I had access to Elders. I had access to medical health practitioners. If I was a, you know, a LGB kid, I knew that there was a LGB resource room, you know, I could go there. I knew where the resources were. But here, it’s just not so apparent, I don’t think.

Considering the lack of support she found in the formal mental health system, Jacinda used self-care strategies to maintain her mental well-being:

I start with some caffeine and a little bit of nutrition and then I go for my daily dose of exercise. It’s all just mental health. Like, I need to do that because I don’t believe in medicating. So I do B vitamins and exercise and that kind of thing. That sort of ensures a good day. If I don’t do it, I’m scattered. Like, I’m just, like, seeds to the wind, man. I’m just all over.

Jacinda also found gardening was a way to address very practical concerns associated with poverty (i.e., food security) at the same time as supporting her mental health:

I think it sort of mitigates some of the depression for sure because I have control of my nutrition, right? And I know where my food is coming from and it helps me feel more grounded in knowing that there’s no chemicals on my food, right. So I really—it’s empowering. And I would definitely recommend to anybody that struggles with poverty or—doesn’t matter if you struggle with poverty—but if you have any sort of issues with mental health, try and grow something because it’s amazing. Yeah. To say ‘wow, that tomato, I grew that’ you know what I’m saying? It’s, it’s amazing.

When asked what she would ideally like to have in her life at the current moment, Jacinda spoke to both her economic and mental health needs:

I would love to be able to access more meaningful employment, that will pay me a living wage. And that’s really hard to come by out here. And if you talk to anyone else, I dunno if that’s going to be their experience. Because, you know, depending on your opportunities and things like that. I have no idea. But, yeah, like, access to services would be huge for me. I would love to get some help and maybe some healing around some of my past experiences. It’s just—I guess I’ve just learned how to soldier on, right? ‘Cause what else are you gonna do? Are you gonna just lie in a pool of your own pity?

#### Deborah’s story: “It’s like buying a lottery ticket”

Deborah is a white bisexual trans woman who was in her late 40s at the time of our interview. In the year prior to meeting with us, Deborah had multiple psychiatric hospitalizations for suicide attempts, which she connected to family (and subsequent economic) crisis that was precipitated when her wife “asked me after 23 years of marriage if I would either stop being transgendered or leave.” Deborah felt that her options were either suicide or leaving her marriage, so she opted to move away from her community—which required closing her self-run business—and start a new life in a major urban centre in Ontario:

I found an apartment in [city] and started to re-establish myself in [city]. And I quickly wasn’t coping very well. I wasn’t able to get on my feet and that’s when the thoughts of suicide got worse. I was hospitalized in July of 2009 and then when I came out of the hospital I ended up in the [crisis center] and then from there a homeless shelter.

In the years since coming to the city, Deborah had a number of mental health hospitalizations, but the care she received there did nothing to address the losses she had experienced as a result of her gender transition or the poverty that was her daily reality.

They’re [mental health providers] not so much worried about the details. They just want to create a category for you and I don’t think transgendered even is in–maybe it is, I don’t think it is–it’s more or less–are you bipolar? Is it situational depression? Is it borderline personality disorder? Is it post traumatic stress? Like, those have nothing to do with gender.

Yet for Deborah, the connection between her trans identity and her mental health could not be more explicit:

That was another time I went to the hospital. Was when the conservatives [the “Institute for Canadian Values”] put an ad in the paper–the ‘please don’t confuse me’ ad campaign [in protest of including information about gender identity in a new sex education curriculum]. Full page ads about Two-spirited people, transgendered people. Anywhere else that would be a hate crime. If you would have inserted any other sector of society, it would be a hate crime. But because it was transgendered people, it was totally acceptable.

Deborah’s story clearly illustrates the work of finding affordable, accessible, and most notably, trans-affirming mental health services, particularly when in crisis:

In July of 2009 I went to [hospital] for the first time. I don’t remember very much of it. I just know I was very confused. I ended up becoming very angry with the way I was treated. I was formed [involuntarily admitted or held]–prior to becoming angry I didn’t know what being ‘formed’ meant. And so, I went in voluntarily and within three days I could start to feel that I was losing control. That I had no, my opinion didn’t matter. I had a doctor who was very set on a diagnosis that involved medication that I didn’t understand…I didn’t react very well once out of the hospital. I ended up trying to get into the [crisis centre]. I could not get through on the phone line. It took me a few days before I got in. I left the hospital on a Thursday and I got into the [crisis centre] on Monday night. I ended up actually going to [crisis centre] and knocking on their door, trying to get in. They were reluctant to let me in. They did let me in. From there I ended up getting into a shelter… I went to a shelter that wasn’t so good. It was very hostile and I stayed there for about a month.

This rotation between hospitals and shelters without any improvement in her mental health had a major impact on Deborah, and just staying alive was a constant struggle:

I didn’t know what to do–three years have gone by of in and out of the hospital–and I was getting really tired of this—showing up to the hospital and going back home again. It just seemed like I was wasting a lot of time and resources where nothing was changing … Every day you think of suicide because you’re tired. On the one hand you don’t want to wait anymore, but then you also think maybe it’s going to be better tomorrow. Maybe something tomorrow will change and someone will figure out what’s wrong and you’ll actually enjoy living again, but it’s a gamble you take over and over and over again. And it never happens–it’s like buying a lottery ticket. The chances of getting diagnosed and getting treated for whatever is happening between your ears—it’s really hard and new for me.

Deborah was able to articulate what it was about the medical care she received in hospital that was unhelpful–and unsafe- for her:

I think having a little bit of control makes [services] safe or if you’re able to express your opinion or your needs–I know a lot of times when you’re dealing with professionals they have a protocol they follow, and you’ve gotta fit their protocol, whereas the place that feels safe to me–they adjust to who I am, and to where I am. So, I guess if I go to some place where I don’t know what their expectations are of me or what that protocol–they may know what’s going on, but I don't.

Deborah’s experience was that her trans identity did not fit this ‘protocol’ that professionals were following:

They would refer to me as [given (male) name] or Mr. [last name]… It’s the sort of thing where it’s very subtle, but it’s just an indication that it’s what’s on your OHIP [provincial health insurance] card that matters, not who you are… Like when you’re sitting in emergency looking like this [female gender presentation] and they call out [given name] or Mr. [last name], and you stand up and there’s people sitting there all around you, you feel weird. You almost don’t want to be in the emergency department, right?

This misgendering in mental health services even extended to the gender identity clinic that Deborah tried to access (“the first thing that happened was the letter that I got that said to come to the appointment was mailed to a women’s shelter, addressed to Mr. [last name]”) and also to the crisis centre Deborah accessed:

[Crisis centre]–they make a big issue about which bathroom you use. I’m going to [college] right now and nobody cares which bathroom I use, you know what I mean? I’m in school with thousands of people and it’s not a problem. You go to the bathroom, you go to the bathroom. But at [crisis centre] there’s nine people in there and–‘Well we’re not sure they can take you because we don’t know how to deal with the bathroom.’

Deborah went on to elaborate her frustration with this transphobic treatment:

And that’s the sad part is, is when you go to emerg or you go to the gender clinic, or you go to the crisis centre. That’s one time–if ever you need extra care, you need someone to just not say those things or not treat you like that. Even if they’re thinking it, don’t verbalize it? Like sometimes it would be nice just to rest and even let your guard down and actually feel safe… I can’t speak for them [mental health providers], but I’m thinking the fact that I’m coming in as transgendered is the biggest hurdle, to me, dealing–we’re not dealing with the triggers for mental health or depression, we’re dealing with the fact that she’s not wearing pants, you know?

The care that Deborah found most helpful to her was outside of the health sector altogether; care that did address the losses she had experienced related to her gender transition and her poverty:

I think the most helpful at that time was my church. They had a depression initiative—they had deacons that started a peer support group and worked with us through dealing with depression… there was one deacon in particular and she was always available. She gave me her cell phone number and her home phone number, and I would check in almost daily to make sure–just to let her know I was okay. We would talk through a lot of stuff…And she also made her–she has a bed and breakfast–made her place available if I needed to get out of the city to just spend a day, you know, in the garden or whatever and just not deal with the stress of the shelters and all.

Deborah also spoke about the value for her in being involved in local support services by and for trans people:

We do have a trans support group at [LGBTQ community centre]. That meets twice a month, but that one’s not so much about mental health. It’s more about—you’re able to talk about what’s happened since the last time you came. It’s almost like an AA meeting where you talk out things. People share resources and leads and stuff….Through that I actually did join the–there’s a team there that does outreach and we do educational stuff. We do workshops… we train service providers to use non-oppressive trans-, LGBTQ-language and go through definitions and scenarios where they can empathize with what it’s like for LGBTQ people… So, it’s sort of–on the one hand we’re going through all these agencies as clients, and on the other hand we’re giving feedback through these workshops to show what it’s like as a trans person. ‘Cause even accessing shelters we’re pretty unique among people that go to shelters. Trans people are sort of a special asterisk at the end of shelter because most shelters don’t accept trans people. So, we try to get more shelters to be inclusive.

This advocacy work was not only personally satisfying for her (“when I do workshops it’s invigorating…I have a sense of accomplishment”), but also an opportunity for employment:

I was self-employed, I had a business. So, I wasn’t able to go back into [home region], so I lost my income. And most of the people that are in the [trans advocacy] program don’t have a job, and so this is a way to get a little bit of part time work.

Deborah also found community in the Occupy movement, which held a major demonstration during this period, and provided a brief respite from her mental health struggles:

And then in October I joined Occupy [city], which got me focused on–it almost created like a family. So, I was not as–I wasn’t suicidal actually. I was coping pretty good, and then we got evicted out of the park. And then for a while we were okay, but by February, I was suicidal again.

In her interview, Deborah reflected on what it was about her experience with Occupy that had such a positive effect on her mental health:

I had purpose. I was with logistics. So, we were constantly doing things. So they would say we needed to do this. I’d say “okay can I handle that”. I would do it… I think that busyness and also being able to identify where the hazards were, like I knew what was safe and what wasn’t safe. Outside of the park I didn’t know what was safe.

Here too, Deborah’s sense of safety was explicitly connected to recognition of her identity as a trans woman:

And that’s another thing within the park too, is people respected me as Deborah. I wasn’t judged for being transgendered. In fact a lot of transgendered people came there because it was a safe place to be. So, I think there was a community or family created within the park where we were like-minded. We had a common goal. We each wanted to be able to tell our story of why we don’t like the system, and then we had people that would empathize and help us create change. And so, it was very empowering, you know what I mean? … So I think that gave me purpose. That gave me family. That gave me some kind of empowerment and in the process of that I was not isolated or suicidal.

## Discussion

In the quantitative strand of this mixed-methods analysis, we identified disparities in mental health service utilization at the intersection of LGBTQ identity and poverty, wherein low income LGBTQ people were more likely than other participants to access professionals (both in terms of any access and in terms of number of professionals seen in the past 12 months) yet simultaneously were less likely than other participants to have their mental health needs met (as indicated by higher reports of unmet need and a lower proportion of professionals seen who were helpful). Our qualitative findings help to explicate this contradiction, through illustrating the extensive work that low income LGBTQ people need to do in order to take care of themselves in the context of a system that largely fails to meet their needs. Specifically, LGBTQ people living in poverty must work against intersectional oppressions on multiple levels: in seeking and retaining employment, in identifying and accessing restrictive low- or–no cost service options, in encounters with discriminatory service providers, and in the day-to-day demands of meeting basic physical and emotional needs with few resources. These encounters with intersectional oppressive systems result both in increased need for wellness supports and in a lower likelihood of having needs met in formal mental health care, for LGBTQ people living in poverty relative to people of other sexual orientation, gender identity, and socioeconomic groups.

Our quantitative findings are consistent with an extensive body of literature that has reported elevated rates of mental health service utilization among sexual minority people relative to heterosexuals [33], but departs from literature suggesting either equivalent or lower rates of mental health service utilization among low income, relative to higher income, people [34–35]. At the same time, however, available data indicate that low income people are over-represented among frequent users of the health care system more generally (i.e., in studies that have not specifically investigated mental health care) [36]. This difference could be at least partially attributable to differential access to general vs. specialist mental health care for low income people [37]: in Ontario’s provincial health insurance system, most specialist mental health care, other than that provided by psychiatrists, is delivered in the private sector and as such is largely inaccessible to those without private health insurance. However, this discrepancy could also be at least partly attributable to differential measurement of mental health service utilization, wherein the majority of studies reporting no difference or lower rates drew from administrative data, while our study drew from self-report data. In this regard, it is likely that our self-report data capture not only “successful” mental health encounters that would be recorded administratively, but also “unsuccessful” attempts at service access, such as telephone or other contacts with services that did not ultimately result in a formal assessment or treatment encounter. In this regard, our self-report data likely more closely reflect individuals’ perceptions of their various (attempted) engagements with mental health systems.

Our work also extends existing literature to demonstrate a further increase in mental health service utilization at the intersection of LGBTQ identity and poverty. In the health services and health policy literature, investigations of high rates of service utilization are often framed around implications for health care spending [38–39], and various interventions have been proposed to decrease utilization among those identified as ‘high cost users’, in order to improve system efficiencies [40]. Our qualitative findings provide essential context to enable us to reconfigure high rates of mental health service utilization among LGBTQ people living in poverty not as a problem to be solved with individual-level interventions, but rather as a problem located within an oppressive mental health system. Indeed, for our participants who had been affected by poverty and violence throughout their lives, the mental health system had never met their needs, and at times had operated (often in concert with other state systems, such as child welfare) as a source of violence and oppression.

As a result, participants in this study described working in resourceful and creative ways to take care of themselves and others in their communities, largely identifying and establishing resources outside of the formal mental health system. The hard work our participants described doing to accomplish taking care of themselves and their communities stands in stark contrast to dominant social narratives about people living in poverty as ‘lazy’, ‘socially disordered’ (i.e., deviant), or ‘cheating the system’ [41]. Our finding that low income LGBTQ people work to create resources and supportive spaces to meet not only their own, but also their peers’ unmet needs similarly stands in contrast to individualist narratives of poor people as undeserving of assistance due to moral or other personal failings [42].

We further this important critique of individualist models through the purposeful integration of an intersectional approach: our data illustrate the ways in which individualist framings of poverty as personal failing are upheld by stigmatization of LGBTQ identities and vice versa, resulting in mutually reinforcing forms of oppression that have implications for mental health. For example, our participants’ narratives illustrate pathways through which discrimination on the basis of LGBTQ identity (e.g., in employment) can make people poor, wherein internalization of individualist models lead to feelings of worthlessness and ‘uselessness’. Individualist models fail to acknowledge that discrimination interferes with LGBTQ (and other oppressed) peoples’ capacity to thrive in a free market (e.g., employment discrimination, disruptions to education/employment associated with mental distress). At the structural level, class-based discrimination and inadequate social policy to address poverty disproportionately affect LGBTQ people, as a result of their increased likelihood of living in poverty [5]. Our data thus provide one further illustration that individualist framings of poverty, while problematic for societies at large, are particularly inapplicable to LGBTQ people and others who experience social oppression. Indeed, our findings illustrate the mechanisms through which inadequate and oppressive public sector mental health services can serve to reproduce and sustain mental health inequities.

The prominence of violence against women and trans people in these participants’ narratives also needs to be considered in research on poverty and mental health care access. This violence was often connected to childhood experiences of living in poverty, and to being part of racialized and transgender communities, and participants described the daily work they needed to do to manage the impact of these experiences. For example, the high rates of violence targeting Indigenous women in Canada, including the multigeneration impact of residential schools, can be seen throughout Jacinda’s account of trying to find safety and support for herself and her daughter. Further, harassment and abuse were sometimes experienced from mental health providers directly. Such cumulative experiences of violence added to the work that participants described having to do as they tried to take care of themselves and their loved ones, and create needed supports outside of formal systems.

The large sample size and ample representation of groups that are often under-represented in LGBTQ health research (people living in poverty, people of colour) are significant strengths of this study. In addition, the mixed methods design enables a more complete conceptualization of the relationship between LGBTQ identity, poverty, and mental health service access than would a mono-method design. Our study also contributes to the field of mixed methods scholarship, by simultaneously providing an example of three approaches that have been under-utilized in mixed methods research to date: reuse or secondary analysis of data; qualitatively-driven mixed methods (as defined using Mason’s [22] original emphasis on broad application of qualitative epistemologies and methodologies); and narrative analysis as the methodological approach for our priority qualitative strand.

However, some important limitations of our work should be noted. First, population-based sampling was not feasible for this study, and as such, we are unable to determine to what extent our quantitative sample reflects the broader population of LGBTQ people living in poverty. With respect to the qualitative strand, the fact that poverty was not an *a priori* focus of the qualitative interviews, nor was narrative analysis envisioned at the time of interviewing, may have resulted in less depth of exploration of relevant topics in interviews than might otherwise have been possible. Our findings cannot be extended to sexual minority and cisgender heterosexual men; further research examining these gender groups is warranted. Similarly, our qualitative analysis focused on participants aged 22–53 years; younger and older LGBTQ people living in poverty may experience quite different encounters with the mental health system. Finally, our work has centered on poverty, yet our qualitative data suggest that other indicators of socioeconomic status (e.g., education, employment) are related in complex ways to LGBTQ peoples’ experiences of both poverty and mental health service experiences. Additional research examining a variety of indicators of socioeconomic status would be warranted.

However, despite these limitations, this study offers an important addition to our very limited understanding of how poverty and LGBTQ identity intersect to determine mental health service experiences. Of note, our findings emphasize that while barriers to service access clearly exist for low-income LGBTQ people, simply removing these barriers (i.e., increasing access) will not be sufficient to create an equitable mental health system. Even once accessed, available mental health services are inadequate to meet the specific needs of LGBTQ people living in poverty, predominantly due to a) a lack of service provider knowledge and preparedness to address issues relevant to LGBTQ people, poverty, and the intersection of these experiences; and b) a dominant biomedical framework that fails to acknowledge the fundamental impact of social discrimination on mental health for poor LGBTQ, and other marginalized, communities. As Mills [43] has noted, dominant biomedical discourse of the inter-relationships between poverty and mental health has been highly successful in reconfiguring the real distress that poverty produces into ‘symptoms’ of ‘mental disorders’, which in turn must be addressed through individual-level interventions to manage distress, rather than through structural level interventions to address the root cause of poverty. Our participants’ narratives illustrate how this focus on individual ‘disorders’ results in a mental health system that serves to reproduce and sustain mental health inequities experience by low income LGBTQ people and other groups living at the intersection of structural oppressions.

## Supporting information

S1 Appendix Quantitative survey measures for a qualitatively-driven mixed methods analysis of the mental health service experiences of LGBTQ people living in poverty in Ontario, Canada. (DOCX)
