# Supplementary material for: In spite of the system: A qualitatively-driven mixed methods analysis of the mental health services experiences of LGBTQ people living in poverty in Ontario, Canada
Source: PLoS One. 2018 Aug 15;13(8):e0201437. doi: 10.1371/journal.pone.0201437 (PMC6093609; doi:10.1371/journal.pone.0201437)
Supplement: S1 Appendix — (DOCX) [file pone.0201437.s001.docx]

Items to assess sexual orientation and gender identity

**How do you describe your gender?** Check all that apply**.**

I identify as a woman

I identify as a man

I identify as trans woman

I identify as trans man

I identify as a woman of trans experience

I identify as a man of trans experience

I identify as genderqueer

I identify as Two-Spirited

I identify as intersex

You don’t have an option that applies to me. I identify as (please specify)

_________________________________________________________

The next question asks about sexual orientation, which is a term for the emotional,

physical, romantic, sexual and spiritual attraction, desire or affection for another person.

**Do you think of yourself as**, (*Check all that apply*)

Lesbian

Heterosexual

Gay

Bisexual

Two-Spirited

Homosexual

Pansexual

Asexual

Queer

You don’t have an option that applies to me. I identify as (please specify)

_________________________________________________________

Items to assess low income

The Low Income Measure (LIM) was calculated according to Statistics Canada instructions against the median income from the 2011 National Household Survey using the following survey items:

1. Household income: *“What was your combined household income before taxes last year?”* Participants were asked to select one of 8 income ranges; the mid-point of the income range was used in LIM calculations.
2. Household size: Responses to the question *“How many people are supported by your household income, including yourself?”* were treated as a continuous variable in LIM calculations.
3. Sources of income: participants were asked to select all that apply from a listing of 9 potential sources of income (e.g., paid employment, disability benefits). For five participants who had missing data for either household income or household size, source of income was used to impute the LIM, wherein participants who reported income supports (i.e., employment income, disability support) as their only source as income were coded as below LIM (in Ontario, income supports provide an annual household income well below the LIM).
4. Proportion of monthly income spent on housing: This was either calculated on the basis of participants’ self reports of both amount spent on housing per month and household income, or determined based on a question asking what proportion of their monthly income was spent on housing (three fixed options: 0-29%, 30-49%, 50-100%). For 12 participants with missing data for either household income or household size, proportion of income spent on housing was used to impute the LIM, wherein participants who reported spending 50-100% of their monthly income on housing were coded as below LIM.

Items to assess mental health service utilization variables

1. Access to mental health services in the past 12 months: *“During the last 12 months, have you seen, talked on the telephone or communicated by internet or email to a professional about your emotions or mental health?”* Responses to an analogous question with a lifetime recall period were forward filled to create a binary (yes/no) variable for past 12 months service access.
2. Unmet need for mental health care in the past 12 months: We created a binary (yes/no) variable on the basis of the question, *“During the past 12 months, was there ever a time when you felt that you needed help from any kind of support for your emotions or mental health but you didn’t receive it?”*
3. Professionals seen in the past 12 months: We created a continuous variable on the basis of the question, *“During the last 12 months, how many professionals have you seen about your emotions or mental health?”*
4. Proportion of professionals seen in the past 12 months who were helpful: We calculated this on the basis of responses to the question “*How many of the professionals that you have seen in the past 12 months did you find helpful?”* divided by the total number of professionals seen in the past 12 months.
